# On the Calibration of GNSS-Based Vehicle Speed Meters †

**DOI:** 10.3390/s20030591

**Published:** 2020-01-21

**Authors:** Adolfo Martucci, Giovanni Cerasuolo, Orsola Petrella, Marco Laracca

**Affiliations:** 1 CIRA (Italian Aerospace Research Centre), Via Maiorise, 81043 Capua, Italy; a.martucci@cira.it (A.M.); g.cerasuolo@cira.it (G.C.); o.petrella@cira.it (O.P.); 2 Department of Electrical and Information Engineering, University of Cassino and Southern Lazio, Via G. Di Biasio 43, 03043 Cassino, Italy

**Keywords:** GNSS, uncertainty, speed measurement, calibration, metrological traceability

## Abstract

Thanks to their metrological characteristics (accuracy, dimensions, synchronization capability, easy interfacing, and so on), in the last few years, the GNSS (Global Navigation Satellite System) based speed instruments are often used in a wide field of application. The traceability of the measurement results achieved by the GNSS instrument should be made by means of calibration procedures in compliance with the ISO/IEC 17025 standard and ILAC (International Laboratory Accreditation Cooperation) policy on the traceability of measurement results. In this context, some calibration methodologies have been proposed in the literature or used by some calibration centers. In a speed range from 1 to 300 km/h, an analysis on the suitability of the experimental calibration method (based on a couple of photocells placed on the road at a certain distance) for the GNSS speed measurement systems is presented in this paper. An analysis of the measurement setup has allowed for the recognition of both all the uncertainty contributions and defines the variability range of their values. After the formulation of the relationships between the uncertainty contributions and the total calibration uncertainty due to the calibration method, the sensitivity analysis has been made. The analyzed measurement setup, even if considering a careful choice of both instrumentations and methodologies, is suitable for the calibration of high accuracy GNSS based instruments only considering distances between the photocells sufficiently large and for speed values lower than 200 km/h. In any case, the proposed analysis can be a useful tool to allow for the choices on the measurement setup to reach the desired trade-off between calibration costs and compliance with technical requirements and also the calibration of instrumentation different by GNSS.

## 1. Introduction

The Global Navigation Satellite System (GNSS) provides autonomous geo-spatial positioning with global coverage. With the GNSS, by means of electronic receivers, it is possible to calculate the position (latitude, longitude, and altitude) with an accuracy ranging from the order of meters up to centimeters, thanks to the use of the signals transmitted along a line of sight from radio to satellites. The first GNSS system comes from a project that was started by the United States of America Department of Defense in the 1970s and was called Global Positioning System (GPS). In fact, the Russian GLONASS is also fully operative, while the Chinese BeiDou Navigation Satellite System (BDS) and the European GALILEO are scheduled to be completed by 2020.

GNSS is largely used in several applications, in particular navigation and speed measurement, thanks to the good accuracy achieved, easy operations, and synchronization [1,2,3,4,5,6,7,8]. Indeed, in the last years, in the field of traffic speed measurement, thanks to the accuracy claimed by the manufacturer of GNSS receivers (about 0.1 km/h, corresponding in an uncertainty of about 0.06 km/h for a uniform distribution), their use as reference equipment for the calibration of speed measurement instruments are increasing [9,10,11,12].

With an aim to ensure the measurement traceability to the International System of Units, the GNSS must be calibrated according to the rules of the ISO/IEC 17025 standard [13] and comply with the ILAC (International Laboratory Accreditation Cooperation) policy on the traceability of measurement results [14]. ISO/IEC 17025 states that “When using external calibration services, traceability of measurement shall be assured by the use of calibration services from laboratories that can demonstrate competence, measurement capability and traceability”. Additionally, for equipment and reference standards that must be calibrated, the ILAC policy states that they shall be calibrated by:

(a) A National Metrology Institute (NMI) whose service is suitable for the intended need and is covered by the CIPM MRA (International Committee for Weights and Measures—Mutual Recognition Arrangement). CIPM MRA is the framework through which National Metrology Institutes demonstrate the international equivalence of their measurement standards and the calibration and measurement certificates they issue.
(b) An accredited calibration laboratory whose service is suitable for the intended need and the Accreditation Body is covered by the ILAC Arrangement or by Regional Arrangements recognized by ILAC.
(c) An NMI whose service is suitable for the intended need but not covered by the CIPM MRA.
(d) A calibration laboratory whose service is suitable for the intended need but not covered by the ILAC Arrangement or by Regional Arrangements recognized by ILAC.

Laboratories that have demonstrated the traceability of their measurements through the use of calibration services offered according to (a) or (b) above have made use of services that have been subject to relevant peer review or accreditation. In the situation where (c) or (d) applies, this is not the case, so these routes should only be applicable when (a) or (b) are not possible for a particular calibration. The laboratory must provide evidence for the claim of measurement traceability and measurement uncertainty and then the accreditation body shall assess this evidence.

In this context, the speed calibration of GNSS equipment ranks in the cases (c) and (d); the cases (b) and (d) are not right because the accredited calibration laboratory [15,16] cannot assure a Calibration and Measurement Capabilities (CMC) appropriate to calibrate high accuracy GNSS.

For example, the Swiss NMI, METAS, falls in the case (c) and it usually calibrates GNSS by means of satellite simulators [17,18], which therefore does not take into account many problems that can be present in only experimental workings [19,20,21,22].

In view of the above limitations, the need to develop a calibration methodology is evident, for vehicle speed measurement GNSS-based instrumentation, to be able to use calibration standards that can grant measurement traceability covered by the CIPM MRA. Some proposal to calibrate or to test the speed measured by GNSS receivers have been made in References [23,24,25,26,27,28], but the determination of a calibration method to be able to grant the desired CMC in a wide speed range, considering all the measurement uncertainties contribution is still missing.

The aim of this work is to analyze the suitability of an experimental calibration method for GNSS speed measurement systems. Among all the solutions presented in References [23,24,25,26,27,28], in this paper, the attention is focused on the calibration methodology presented in References [23,24] and is based on the use of two pairs of photocells on a track together with a frequency counter and a synchronization system. Considering all the measurement uncertainty contribution, a sensitive analysis has been made in order to understand the limits and/or the applicability of this calibration methodology in a speed range from 1 to 300 km/h. A preliminary study in this direction has been presented in Reference [29].

## 2. The Considered Measurement Setup

**Figure 1:**
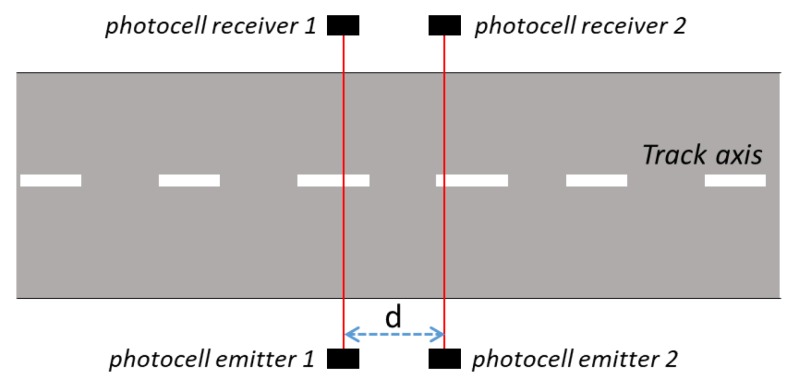
A schematic of the two couple of photocells positioned on the track.

The reference measurement set-up takes into account a system of photocells for the measurement of the speed of a vehicle on which is installed the GNSS based speed meters under calibration. In Figure 1, “*d*” is the distance between the two couples of photocells along the way traveled by the vehicle.

**Figure 2:**
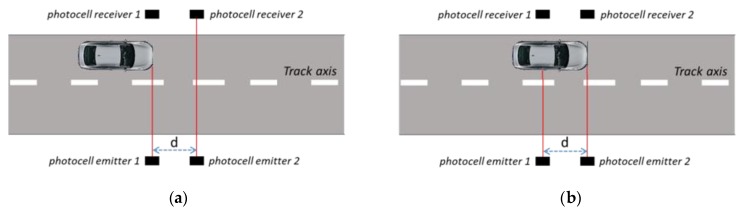
Representation of the vehicle that is seen by the first (**a**) and second (**b**) photocells couple.

Indicating with *T*, the time interval taken by the car to travel the distance “*d*” (passage between the situation of Figure 2a to the one of Figure 2b), the speed of the reference system can be evaluated as:

(1) $$V_{ref}=\frac{d}{T}$$

The time interval *T* can be measured by means of an electronic counter connected to the start (situation of Figure 2a) and stop (situation of Figure 2b) signals, generated by the photocells couples.

The typical output of a calibration process is the evaluation of the absolute error (∆V) and its corresponding uncertainty ($u_{∆V}$) starting from the measured quantities (the speed of the vehicle in the considered case) made by the reference instrument (*V_ref_*) and by the instrument under calibration (*V_UUC_*).

(2) $$∆V=V_{UUC}-V_{ref}$$

The value of *V_UUC_* is obtained as the average of the N speed ($V_{UUC_{i}}$) measured by the GNSS device during the time interval *T*.

(3) $$V_{UUC}=\frac{1}{N}\sum_{i=1}^{N}V_{UUC_{i}}$$

The number of measured speeds (*N*) depends on the update rate of GNSS device and on the time interval *T* (that is strictly related to the distance “*d*” and on the speed of the vehicle for the specific testing point).

In the proposed solution, since the measure of *V_UUC_* is made on the vehicle while the measure of *V_ref_* is made on the ground, both a suitable choice of the distance between the photocells and a suitable synchronization method must also be considered for the comparison of these two speeds (to be sure that both the reference system and the instrument under calibration measure the vehicle speed in the same time and in the same place).

As far as the distance between the photocells is concerned, it is necessary to observe that, typically, GNSS receivers are characterized by update rates from 1 Hz to 100 Hz. This means that the output data of the GNSS system (position, speed, etc.) are updated with a time interval “*t_i_*” from 1 to 0.01 s. Considering a vehicle travelling at speed “*v_i_*”, the speed measured by a GNSS receiver (installed on board) is made at a distance interval $d_{i}=v_{i}\cdot t_{i}$. As it is possible to see in Table 1, the distance interval between two consecutive measurements made by the GNSS receiver changes from 3 mm to about 83 m. This means that the distance between the photocells cannot be fixed and its choices must be made appropriately to be sure that at least one point ($V_{UUC_{i}}$) of the speed measured by the GNSS device is made during the time interval *T* (in other words, inside the space between the two photocells).

These considerations have a direct impact on the possible choices that can be made in the installation of the two photocells couples, and consequently, in the uncertainty contributions to the knowledge of the distance “*d*” (so, on the uncertainty of $V_{ref}$). In fact, if “*d*” is fixed and less than about 3–4 m (for transportability purposes), a fixed installation of the photocells on a bar can be hypothesized. In this way, the distance “*d*”, the height of the photocells from the bar, the photocells orientation with respect to the bar, etc., can be characterized and known with a suitable uncertainty by using laboratory equipment. Otherwise, the photocells can be installed on the ground using adjustable tripods. In this case, the installation is more flexible but also more complex and the measurement of “*d*”, the installation height of the photocells from the ground, the photocells orientation respect to the track, etc., must be made on site and by using in field instrumentations.

As far as the synchronization system is concerned, it has a direct effect on the calibration uncertainty due to the not stable speed of the vehicle during the tests.

Two possible solutions can be adopted for the synchronization purpose.

(i). The first one is based on the use of a device to be able to communicate to the GNSS receiver (UUC) the start and stop instant times. This can be made, for example [12,13], by means of two flashlight emitters, placed on the ground, that are triggered correspondingly with the start and stop instant times and two flashlight receivers, positioned on the vehicle, that sense the start and stop instant times allowing for the creation of start and stop flags in the speed values acquired by UUC. The application of this solution is conditioned by the availability of a GNSS device equipped with a synchronization data input port or the availability of an additional central unit to be able to acquire, simultaneously, both the speed values coming from the UUC and the synchronization data coming from flashlight receivers.
(ii). The second one is based on the use of UTC time (Coordinated Universal Time). Since any GNSS system always furnish the measured data (e.g., the speed) accompanied by corresponding UTC time, the idea is to use a GNSS receiver with a high update rate (e.g., 100 Hz), and equipped with a synchronization data input port. It is positioned on the ground and connected with the photocells. In this way, it is possible to create flags in the UTC time registered by the GNSS receiver triggered with the start and stop instant times. The flagged UTC times, stored by the GNSS system on the ground, are then used for the synchronization with the speed values measured by the UUC.

The solution (i) gives rise to an important limitation: It is not possible to calibrate GNSS devices that are not equipped with a synchronization data input port or it is not possible to acquire the measured data by means of an additional central unit system. In addition, there is a calibration procedure complexity due to the need to create ad hoc interfacing with each UUC to give signals on the synchronization data input port (with different kind of signals, voltage levels, etc.) or to acquire the data from their output port (different hardware, transmission protocols, data format, etc.). For this reason, the synchronization solution (ii) is preferable and is chosen in the analysis made in this paper.

## 3. Analysis of the Uncertainty Contributions

The main scope of this paper is referred to as the analysis of the uncertainty contributions of the calibration setup shown in Section 2 for the calibration of GNSS based speed meters.

Considering all the influence quantities in the evaluation of the calibration error ∆V, Equation (2) can be rewritten as:

(4) $$∆V=(V_{UUC}+∆V_{UUC})-(V_{ref}+∆V_{ref})+∆V_{SYNC}$$

Some of these uncertainties contributions are strictly connected to the instrument under calibration ($∆V_{UUC}$ related to the measure of *V_UUC_*), others are connected to the considered calibration method ($∆V_{ref}$ related to the measure of *V_ref_*, and $∆V_{SYNC}$ related to the synchronization capability between *V_ref_* and *V_UUC_*).

$∆V_{UUC},\;∆V_{ref}$, and $∆V_{SYNC}$ can be considered at a zero mean value and with uncertainty $u_{∆V_{UUC},\;}u_{∆V_{ref},\;}\mathrm{and}\;u_{∆V_{SYNC}}$, respectively. Therefore, taking into account Equation (4), the uncertainty ($u_{∆V}$) of the error ∆V can be evaluated by means of the following equation:

(5) $$u_{∆V}=\sqrt{u_{∆V_{UUC}}^{2}+u_{∆V_{ref}}^{2}+u_{∆V_{SYNC}}^{2}}$$

In the following subsections, a discussion on the abovementioned uncertainty contributions is detailed.

### 3.1. Uncertainty Sources on the Measure of $V_{UUC}$

The influence quantities connected to $∆V_{UUC}$ are due to the metrological characteristics of the device under calibration and can be considered at a zero mean value and with uncertainty $u_{∆V_{UUC}}$.

Defining with $ACC_{UUC}$ the accuracy furnished by the constructor of the UUC and with $RES_{UUC}$ the resolution of the UUC, considering a uniform distribution, $u_{∆V_{UUC}}$ can be written as:

(6) $$u_{∆V_{UUC}}=\sqrt{{\left(\frac{ACC_{UUC}}{\sqrt{3}}\right)}^{2}+{\left(\frac{RES_{UUC}}{\sqrt{12}}\right)}^{2}}$$

### 3.2. Uncertainty Sources on the Measure of $V_{ref}$

Considering that the speed $V_{ref}$ is evaluated by using Equation (1), the parameters to be considered for the determination of the sources of uncertainty are the distance d and the time interval *T*, from which the uncertainty of the speed $u_{∆V_{ref}}$ can be evaluated by means of the following equation:

(7) $$u_{V_{ref}}=\sqrt{{\left(\frac{\partial V_{ref}}{\partial d}\right)}^{2}\cdot u_{d}^{2}+{\left(\frac{\partial V_{ref}}{\partial T}\right)}^{2}\cdot u_{T}^{2}}$$

where $u_{d}$ and $u_{T}$ are the uncertainties on the knowledge of the distance and of the time interval, respectively. It is clear that to calculate $u_{V_{ref}}$ it is therefore necessary to also consider all the sources of uncertainty that contribute to $u_{d}$ and $u_{T}$.

In the following subsections, a discussion on the uncertainty sources, divided for the contribution on the knowledge of the distance “*d*” and the time interval *T*, is reported.

#### 3.2.1. Uncertainty Sources on the Distance “*d*”

**Figure 3:**
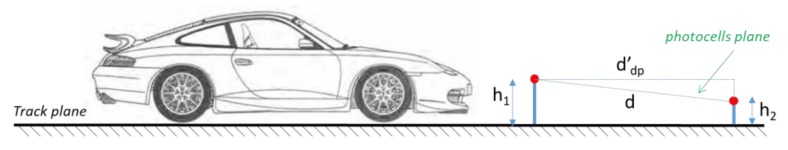
Uncertainty due to the difference in the plane formed by the two couples of photocells with respect to the track plane.

**Figure 4:**
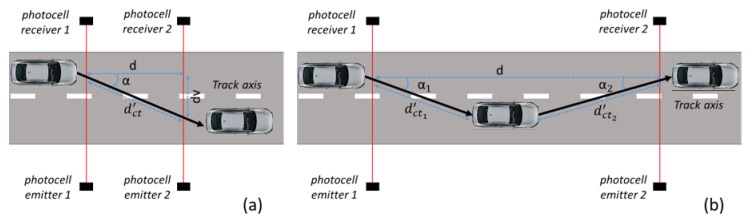
Uncertainty contribution due to the vehicle trajectory along the track: short distances (**a**) and long distances (**b**).

**Figure 5:**
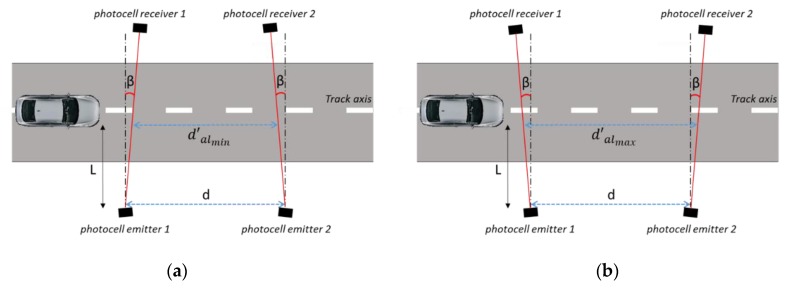
Uncertainty contributions due to the error into the installation of the photocells transmitter-receiver in a direction not perpendicular to the longitudinal axis of the track. Underestimation (**a**) and overestimation (**b**) of the distance between the two photocells laser beams passed through by the vehicle.

**Figure 6:**
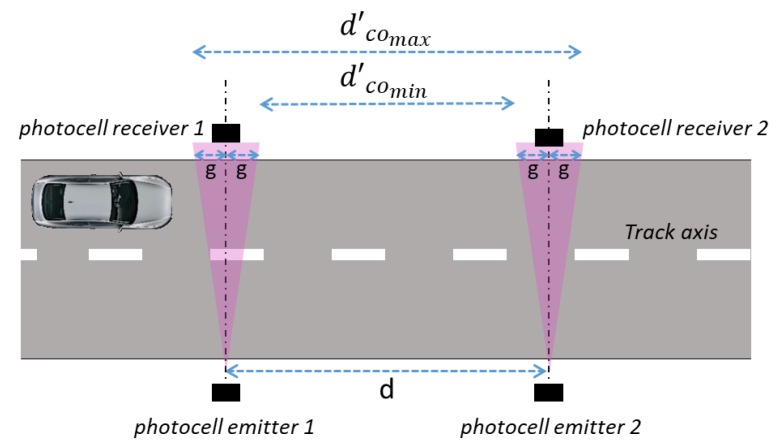
Uncertainty contribution due to the not collimated laser beam.

- Uncertainty of the measure of the distance between the two photocells ($u_{∆d_{ACC}}$). It is connected to the accuracy ($ACC_{d}$) of the instrument used to measure the distance d. Considering a uniform distribution, this uncertainty contribution can be written as:
  (8) $$u_{∆d_{ACC}}=\frac{ACC_{d}}{\sqrt{3}}$$
- Uncertainty in the calibration of the instrument used to measure the distance ($u_{∆d_{CAL}}$). It can be taken from the calibration certificate of the instrument.
- Uncertainty due to the difference in the plane formed by the two couples of photocells respect to the track plane. As is shown in Figure 3, if the installation heights of the two photocells from the road surface ($h_{1}$ and $h_{2}$) are not the same, the distance $d_{dp}^{\prime }$, passed through by the vehicle, is different with respect to the known distance “*d*” between the photocells. The magnitude of this effect is connected to the influence quantities that depend on the choice made for the installation of the photocells couples: On the rod or tripods. Generally, in addition to the accuracy of the instrument used to measure $h_{1}$ and $h_{2}$, possible influence quantities are the asphalt roughness and/or subsidence, track planarity (especially for high values of “*d*”), etc.
  The error, $∆d_{dp}$, in the knowledge of “*d*” can be evaluated as:
  (9) $$∆d_{dp}=d-d_{dp}^{\prime }=d-\sqrt{d^{2}-{\left(h_{1}-h_{2}\right)}^{2}}.$$
  This effect can be considered with a zero mean value and with uncertainty $u_{∆d_{dp}}$ that, considering a uniform distribution, can be written as:
  (10) $$u_{∆d_{dp}}=\frac{∆d_{dp}}{\sqrt{12}}$$
- Uncertainty due to the difference between the vehicle trajectory and the longitudinal axis of the track.
  Considering the schemes shown in Figure 4a, the vehicle could travel along a trajectory that is not perfectly aligned to the track axis (to have a clear draw, the cars positions, and consequently the effects of car trajectory, were amplified with respect to the reality). In this condition, the distance $d_{ct}^{\prime }$ passed through by the vehicle and is different with respect to the known distance “*d*” between the photocells. The error, $∆d_{ct}$, in the knowledge of “*d*” can be evaluated as:
  (11) $$∆d_{ct}=|d-d_{ct}^{\prime }|=|d-\frac{d}{\cos\alpha }|=\left|d\cdot \left(1-\frac{1}{\cos\alpha }\right)\right|.$$
  The amplitude of this effect is strictly connected to the skills of the driver, the value of the distance d, and the speed of the test vehicle. Fixing the driver skills, the greater the distance *d* and the lower the speed value, the greater $∆d_{ct}$ can be. For instance, in Figure 4b, a case in which for longer distance *d* the driver correcting the trajectory around the track axis can have an amplification of the effect on $∆d_{ct}$ is shown.
  This effect can be considered with a zero mean value and with an uncertainty $u_{∆d_{ct}}$ that, considering a uniform distribution, can be written as:
  (12) $$u_{∆d_{ct}}=\frac{∆d_{ct}}{\sqrt{12}}$$
- Uncertainty due to the error into the installation of the photocells transmitter-receiver in a direction not perpendicular to the longitudinal axis of the track.
  As is drawn in Figure 5, the laser beams emitted by the photocells can form an angle (*β*) respect to the direction orthogonal to the track axis. This can be caused by the not ideal methodology used to install of the photocells couples on the track. As a consequence of this effect, the distance $d_{al}^{\prime }$, passed through by the vehicle, ranges from a minimum of $d_{al_{min}}^{\prime }=d-2\;L\;tan\beta$ (condition shown in Figure 5a) to a maximum of $d_{al_{max}}^{\prime }=d+2\;L\;tan\beta$ (condition shown in Figure 5b), where L is the distance from the photocell laser emitters at which the vehicle passes through on the track.
  Correspondingly, the maximum error, $∆d_{al}$, in the knowledge of “*d*” can be evaluated as:
  (13) $$∆d_{al}=d_{al_{max}}^{\prime }-d_{al_{min}}^{\prime }=4\cdot L\cdot \tan\beta$$
  This effect can be considered with a zero mean value and with uncertainty $u_{∆d_{al}}$ that, considering a uniform distribution, can be written as:
  (14) $$u_{∆d_{al}}=\frac{∆d_{al}}{\sqrt{12}}$$
- Uncertainty due to the not collimated laser beam.
  As shown in Figure 6, the laser beams emitted by the photocells could not be perfectly collimated and the presence of the vehicle (start and stop conditions) can be detected in a point that lies inside a cone around the photocells emitter-receiver line. As a consequence, the start and stop conditions can occur in a point upstream or downstream this line, resulting in a variation of the distance “*d*”. The effect increases with the distance of the vehicle from the photocell emitters and the maximum value (*g*) can be taken in the points the photocells receivers are positioned.
  As a consequence of this effect, the distance $d_{co}^{\prime }$, passed through by the vehicle, ranges from a minimum of $d_{co_{min}}^{\prime }=d-2\;g$ to a maximum of $d_{al_{max}}^{\prime }=d+2\;g$.
  Correspondingly, the maximum error, $∆d_{co}$, in the knowledge of “*d*” can be evaluated as:
  (15) $$∆d_{co}=d_{co_{max}}^{\prime }-d_{co_{min}}^{\prime }=4\cdot g$$
  This effect can be considered with a zero mean value and with uncertainty $u_{∆d_{co}}$ that, considering a uniform distribution, can be written as:
  (16) $$u_{∆d_{co}}=\frac{∆d_{co}}{\sqrt{12}}$$
- Uncertainty due to the thermal expansions.
  Since the measurement collection can take several hours and in this time interval the ambient temperature can obviously change, a thermal expansion of the setup components could occur. In particular, there may be a thermal expansion of the rigid bar on which the photocells are mounted (if the tripod solution is chosen to install the photocells on the track, this uncertainty contribution can be neglected). Obviously, the rod should be made of a material with a low coefficient of thermal expansion.
  Indicating with the λ the linear coefficient of thermal expansion of the material taken into account, and with ΔK the maximum temperature difference during the calibration, the error $∆d_{te}$ in the knowledge of “*d*” can be evaluated as:
  (17) $$∆d_{te}=\lambda \cdot d\cdot ∆K$$
  This effect can be considered with a zero mean value and with uncertainty $u_{∆d_{te}}$ that, considering a uniform distribution, can be written as:
  (18) $$u_{∆d_{te}}=\frac{∆d_{te}}{\sqrt{12}}$$
  In conclusion, the distance “*d*” can be expressed by the following equation:
  (19) $$d=d+∆d_{Acc}+∆d_{dp}+∆d_{ct}+∆d_{al}+∆d_{co}+∆d_{te}$$
  and its uncertainty can be expressed by:
  (20) $$u_{d}=\sqrt{u_{∆d_{Acc}}^{2}+u_{∆d_{CAL}}^{2}+u_{∆d_{dp}}^{2}+u_{∆d_{ct}}^{2}+u_{∆d_{al}}^{2}+u_{∆d_{co}}^{2}+u_{∆d_{te}}^{2}}$$

#### 3.2.2. Uncertainty Sources on the Time Interval “*T*”

The influence quantities connected to the measure of the time Interval “*T*” are due to the metrological characteristics of the instrument used to measure “*T*” and the response delay of the photocells. In particular, the time interval “*T*” can be expressed by means of Equation (21)

(21) $$T=T+∆T_{Acc}+∆T_{RES}+∆T_{CAL}+∆T_{rd}$$

where:

- $∆T_{Acc}$ is connected to the accuracy ($ACC_{T}$) of the instrument (e.g., electronic counter) used to measure the time interval *T*. This effect can be considered with a zero mean value and with uncertainty $u_{∆T_{ACC}}$ that, considering an uniform distribution, can be written as:
  (22) $$u_{∆T_{ACC}}=\frac{ACC_{T}}{\sqrt{3}}$$
- $∆T_{RES}$ is connected to the resolution ($RES_{T}$) of the instrument (e.g., electronic counter) used to measure the time interval *T*. This effect can be considered with a zero mean value and with uncertainty $u_{∆T_{RES}}$ that, considering an uniform distribution, can be written as:
  (23) $$u_{∆T_{RES}}=\frac{RES_{T}}{\sqrt{12}}$$
- $∆T_{CAL}$ is connected to the calibration of the instrument used to measure the time interval *T*. The uncertainty value ($u_{∆T_{CAL}}$) can be taken from the calibration certificate of the instrument.
- $∆T_{rd}$ is connected to the response delay of the photocells, and more generally, of the whole electronics used to transform the vehicle passage through the photocells to electronic signals that drive the start and stop condition in the measure of the time interval *T*. Knowledge about this effect can be taken from the manual of the photocells and the used electronics as well as by a metrological characterization of these components. This effect can be considered with a zero mean value and with uncertainty $u_{∆T_{rd}}$ that, considering a uniform distribution, can be written as:
  (24) $$u_{∆T_{rd}}=\frac{∆T_{rd}}{\sqrt{12}}$$
  In conclusion, the uncertainty of the time interval *T* can be expressed by the following equation:
  (25) $$u_{T}=\sqrt{u_{∆T_{Acc}}^{2}+u_{∆T_{RES}}^{2}+u_{∆T_{CAL}}^{2}+u_{∆T_{rd}}^{2}}$$

### 3.3. Uncertainty Sources Due to the Synchronization

**Figure 7:**
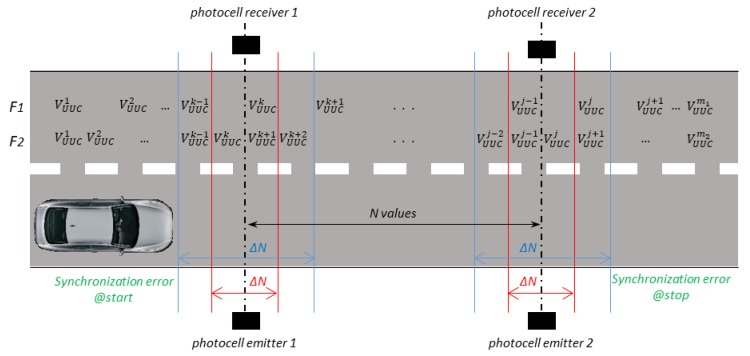
Schematic of the uncertainty contribution due to the synchronization.

As detailed in Section 2, since the measure of V*_UUC_* is made on the vehicle while the measure of *V_ref_* is made on the ground, a suitable synchronization method must be considered for the comparison of these two speeds (to be sure that both the reference system and the instrument under calibration measure the vehicle speed in the same time and in the same place). The GNSS systems measure and store the speed $V_{UUC}^{i}$ with a specific update rate. Figure 7 shows a schematic representation of the speeds measured by the UUC installed on the vehicle while it goes through the reference system based on the photocells. Two update rates ($F_{1}$ and $F_{2}$, with $F_{1}=2\cdot F_{2}$) of the GNSS system is represented.

The speed measured by the reference system ($V_{ref}$, calculated by means of the Equation (1)) corresponds to the mean speed of the vehicle passing between the start and stop situations. Similarly, the speed measured by the UUC ($V_{UUC}$) to be compared with $V_{ref}$ must be the mean value of the *N* speeds $V_{UUC}^{i}$ measured by the UUC between the start and stop situations (see Equation (3)). The number (*N*) of the speeds $V_{UUC}^{i}$ to be considered depends on the vehicle speed, on the GNSS update rate, and on the distance “*d*” between the photocells. In addition, *N* depends on the synchronization capability of calibration method to be used. In fact, an error (see blue and red lines in Figure 7 as examples) in the flagging of the start and stop situations occurs in a change in the number *N* of the speeds $V_{UUC}^{i}$. Obviously, if the vehicle speed is perfectly constant, the mean value does not change. However, if the vehicle speed is not constant, changing *N* also changes the (mean) value of the $V_{UUC}$. The impact of this effect on the uncertainty of the comparison between $V_{UUC}$ and $V_{ref}$ is first connected to the weight of *N* with respect to the variability of *N* (∆N) caused by the synchronization error. For the sake of simplicity, in this paper, we consider $∆V_{SYNC}$ equal to the variability of the speed measured by the GNSS between the start and stop. Since this variability can be obtained from a high number *N* of $V_{UUC}^{i}$ values (high values of both GNSS update rate and *d*), but also from few $V_{UUC}^{i}$ values (low values of both GNSS update rate and *d*) a maximum deviation has been considered rather than a standard deviation to represent the speed variability.

In conclusion, this effect can be considered with a zero mean value and with uncertainty $u_{∆V_{SYNC}}$ that, considering a uniform distribution, can be written as:

(26) $$u_{∆V_{SYNC}}=\frac{∆V_{SYNC}}{\sqrt{12}}$$

where $∆V_{SYNC}=\max{\left(V_{UUC}^{i}\right)}_{i=1}^{N}$ − $\min{\left(V_{UUC}^{i}\right)}_{i=1}^{N}$.

## 4. Variability Range of the Considered Uncertainty Contributions

As is usual in engineering applications, the definition of systems, equipment, and instrumentation, specific installation methodologies to solve a specific problem must be obtained as a compromise between the physical costs of the equipment to be used, the costs of the complexity in the application of the chosen methodology, and technical sustainability of the choices made to ensure that technical constraints are met.

Therefore, once the general lines of the calibration methodology have been chosen (the use of two pairs of photocells and a synchronization system), the technician has a freedom of choice of installation methodology, systems, equipment, the instrumentation to be used, and so on.

For this reason, the uncertainty contributions detailed in Section 3 can change in value depending on the choices made by the technicians. As a consequence, in order to make a sensitivity analysis to be able to highlight the suitability (limits and applicability) of the calibration methodology under investigation, a definition of the variability range (maximum and minimum values) of these uncertainty contributions is necessary.

In Table 2, a summary of the variability range used in the sensitivity analysis is reported, while in the following, a short description of the choices made is detailed.

The variability ranges of the parameters reported in Table 2 have been defined following the considerations reported below.

1. *Speed*—While the GNSS systems are usually able to measure speeds above 1000 km/h, the variability range of the speeds to be investigated using the calibration method under investigation has to be limited to a value allowed by a vehicle that travel on a track with a suitable speed stability. For this reason, a maximum speed value equal to 300 km/h has been chosen.
2. *d*—The choice of the maximum value of the distance between the two photocells couples was made, taking into account the considerations made in Section 2 regarding the distance interval between two consecutive measurements made by the GNSS receiver related to the update rate of the GNSS systems and the speed testing point (see Table 1). Therefore, in order to guarantee that at least one sample of the speed measured by GNSS system is inside the two pairs of photocells for all possible GNSS update rates and a speed up to 300 km/h, the maximum value of d was fixed at 85 m. As far as the minimum value of d is concerned, it has been chosen, taking into account that, typically, reducing d increases the uncertainty. A value equal to 1 m seemed to be sufficiently low to have an analysis with a wide view.
3. $ACC_{d}$—The values reported in Table 2 have been obtained analyzing the typical accuracies of the instruments present on the market for the measure of distance in field. The minimum value has been chosen, taking into account the typical values of their resolution.
4. (h_1_ − h_2_)—As far as the knowledge of h_1_ and h_2_, in addition to the typical accuracies of the instruments present on the market for the in field measure of distance, the effect due to the roughness and the planarity of the asphalt of the track ground surface should be taken into account. The value of $∆d_{dp}$ is obtained by means of Equation (9), starting from the chosen values of h_1_ − h_2_ and d inside the ranges shown in Table 2.
5. α—As described in Section 3, the car trajectory effect and, consequently, the variability range of α is strictly connected to the skills of the driver, to the value of the distance d and to the speed of the test vehicle. The lower the vehicle speed, the greater the possibility to have a slight deviation of the car trajectory with respect to the track axis. At the same time, the possibility to have a car trajectory deviation increases versus the distance d. Obviously, fixing the deviation dv (see Figure 4a), increasing the distance d decreases the angle α, but at the same time, increasing the distance d will increase the possibility of having bigger deviations (also due to the multiple deviation, as represented in Figure 4b). Finally, as shown in Table 2, a variability range from 0 ° to 2 ° has been chosen for the parameter α. In order to take into account the dependency of α with distance d, the following sub-range has been considered:
  - 0° < α < 2° for 1 m < *d* < 5 m (as consequence, considering α = 2°, dv ranges from 3.5 cm to 17 cm changing *d* from 1 m to 5 m respectively);
  - 0° < α < 1° for 6 m < *d* < 20 m (as consequence, considering α = 1°, dv ranges from 11 cm to 34 cm changing *d* from 6 m to 20 m respectively);
  - 0° < α < 0.5° for 20 m < *d* < 85 m (as consequence, considering α = 0.5°, dv ranges from 22 cm to 75 cm changing *d* from 25 m to 85 m respectively)
6. β—The value of this parameter is strictly connected to both the methodology and the instrumentations used to grant the orthogonality among the photocell lasers and the road axis. The maximum value of β is obtained, assuming a basic methodology based on the measure of the distance among the two photocells couples (emitter and receivers) and considering a maximum accuracy in the measure of the distance equal to the maximum value of $ACC_{d}$.
7. $∆d_{co}$—The maximum value of $∆d_{co}$ is obtained by means of Equation (15), considering typical values achieved by the sizes of the laser cone area of commercial photocells for g.
8. $∆d_{te}$—As described in Section 3, the effect due to the thermal expansion is not negligible only in the case of the photocells installed by means of a rigid bar. The maximum value of $∆d_{te}$ is obtained by means of Equation (17), considering the maximum values of λ, d, and ΔK. As far as λ is concerned, the value of the thermal coefficient of the aluminum has been chosen. Considering that the use of a rigid bar is both manageable and feasible for distances d that are not too long, the maximum value of d was set to 10 m. Since the measurements could be made in all year days, a maximum temperature variation ΔK = 40 K has been considered.
9. $ACC_{T}$—There is a wide choice of instruments to be able to measure the time. The corresponding accuracy can vary in a wide range that depends on the cost of the instrumentation. The values of $ACC_{T}$ reported in Table 2 are representative of the instrumentation on the market.
10. $RES_{T}$—Consideration similar to the parameter $ACC_{T}$ can be made.
11. $∆T_{rd}$—The values reported in Table 2 for this parameter are derived by the typical values of the response delay of the photocells available on the market.

## 5. Results and Discussion

Considering the uncertainty contributions discussed in Section 3 and their variability range discussed in Section 4, a sensitivity analysis concerning the influence on the uncertainty of the calibration methodology has been carried out. In particular, this paper is focused on the analysis and the suitability of an experimental calibration method for GNSS speed measurement, and so only the uncertainty contributions connected to the calibration methodology have been taken into account. Therefore, in the present article, the uncertainty connected to the metrological characteristics of the device under calibration ($u_{∆V_{UUC}}$) will be set equal to zero in order to furnish results not dependent on the UUC. Finally, the uncertainty of the calibration method ($u_{∆V_{m}}$) is obtained by Equation (5), setting to zero $u_{∆V_{UUC}}$.

The analysis has been organized, considering fixed values for some of the uncertainty contributions reported in Table 2 and a variation, in their own range, of the parameters under analysis. The fixed values were suitably or randomly chosen inside their own range of variability. In order to allow for an in-depth analysis that considers both a freedom of choice and a large number of combinations of the uncertainty contributions values (inside their defined range of variability), the proposed study has been made in a simulation environment.

### 5.1. Case Study 1

**Figure 8:**
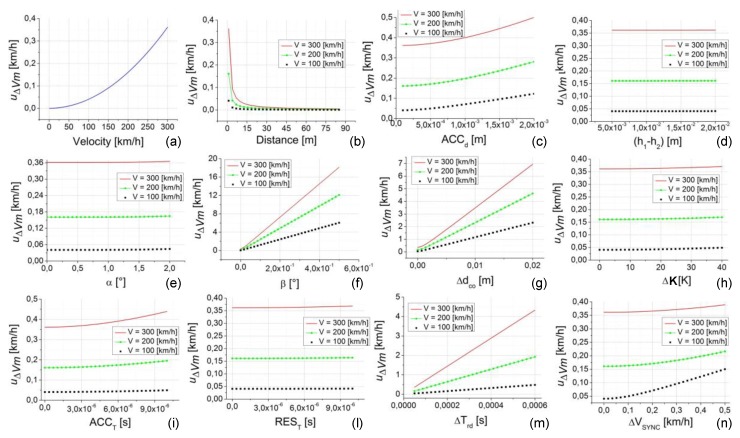
Uncertainty of the calibration method ($u_{∆V_{m}}$) versus the uncertainty contribution parameters. In each plot from (**a**–**n**) the independent variable is one parameter. The plots are relative to the case study 1 (d = 1 m, speed = 100, 200, and 300 km/h, all the other parameters are set to their minimum value, as reported in Table 2).

The analysis of the first considered case study is reported in Figure 8. Except the speed, all the uncertainty contribution parameters are fixed at their minimum value (considering their own range of variability reported in Table 2). The analysis is repeated for three values of speed (100, 200, and 300 km/h), except for the data shown in Figure 8a, since the speed is the parameter under investigation so in that case it varies in all its variability range. The distance d is fixed to their minimum value (1 m) in all the analyses, except the data shown in Figure 8b since, in this case, d is the parameter under investigation so it varies in all its variability range.

This case study allows us to highlight the contribution to the uncertainty of the calibration method ($u_{∆V_{m}}$) due to the single parameter (since all the others are set to their minimum value, often zero).

Looking at Figure 8, a number of considerations can be made about the influence of the single parameters to the uncertainty $u_{∆V_{m}}$.

- $u_{∆V_{m}}$ is strongly influenced by the testing speed, varying from 0 to about 0.36 km/h, with the speed range increasing from 1 to 300 km/h, respectively (Figure 8a);
- As shown in Figure 8b, the distance d also has a strong effect on $u_{∆V_{m}}$, especially for lower values of the distance (from 1 to 20 m). The influence is always dependent on the selected speed (100, 200, and 300 km/h) with an increase of $u_{∆V_{m}}$, increasing the speed value. This effect is related on the greater weight of the uncertainty contributions on both the distance *d* and the time *T* when *d* and *T* are smaller (in this case d is at its minimum value equal to 1 m and *T* is small with low values of *d* and high values of speed).
- The influence on $u_{∆V_{m}}$ due to the parameter $ACC_{d}$ is slightly lower with a variation of $u_{∆V_{m}}$ from 0.36 to 0.5 km/h for the case related to 300 km/h and maximum values of about 0.12 km/h and 0.28 km/h for 100 and 200 km/h, respectively (see Figure 8c).
- The effects due to $∆d_{dp}$ ($h_{1}-h_{2}$), $∆d_{ct}$ (α), $∆d_{te}$, and $RES_{T}$ (see Figure 8d,e,h,l, respectively) are all dependent on the speed value (with greater values of $u_{∆V_{m}}$, up to about 0.35 km/h, for higher values of speed), but they give negligible variation to $u_{∆V_{m}}$.
- The effects due to $∆d_{al}$ (β), $∆d_{co}$, $ACC_{T}$, $∆T_{rd}$, and $∆V_{SYNC}$ (see Figure 8f,g,i,m,n, respectively) give significant variation to $u_{∆V_{m}}$ that increases with the increasing of the speed value. Looking at Figure 8f it is possible to note that the effect of $∆d_{al}$ gives rise to a $u_{∆V_{m}}$ greater than 1 km/h already at β values of about 0.1 °, reaching more than 15 km/h for the higher values of speed and β (this effect is connected to the methodology and instrumentation to be used for the calibration setup and it is so high because of the weight of $∆d_{al}$ on the distance d that is fixed to its minimum value).
- Effects on $u_{∆V_{m}}$ greater than 1 km/h (up to about 5–7 km/h) can also be observed for $∆d_{co}$ (Figure 8g) and $∆T_{rd}$ (Figure 8m). These last effects are connected to the choice, the quality, and consequently, the cost of the photocells.

### 5.2. Case Study 2

**Figure 9:**
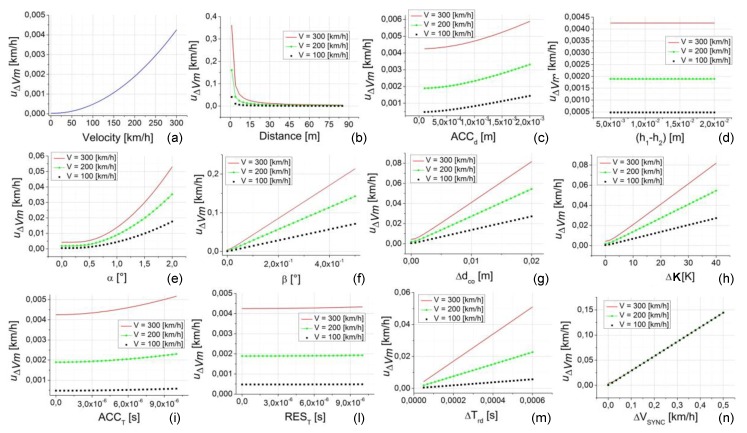
Uncertainty of the calibration method ($u_{∆V_{m}}$) versus the uncertainty contribution parameters. In each plot from (**a**–**n**) the independent variable is one parameter. The plots are relative to case study 2 (d = 85 m, speed = 100, 200, and 300 km/h, all the other parameters set to their minimum value, as reported in Table 2).

The analysis of the second case study is reported in Figure 9. This case study was made in exactly the same conditions of case study 1 with the only difference being that the distance d was fixed at its maximum value equal to 85 m (except that for the data shown in Figure 9b where *d* is the parameter under investigation so it varies in all its variability range).

This case study allows us to highlight the contribution to the uncertainty of the calibration method ($u_{∆V_{m}}$) due to the single parameter (since all the others are set to their minimum value, often zero) and when the distance d is set to the maximum value of 85 m (that is the value in which its contribution to the uncertainty is minimum). Thus, this case study can be seen as concerning the minimum reachable uncertainty values.

Looking at Figure 9, a number of considerations can be made.

- The first general consideration is that, thanks to the high value of *d*, all $u_{∆V_{m}}$ values are heavily lower than the case study 1.
- Obviously, Figure 9b is the same as Figure 8b so the considerations are the same.
- The effects due to speed, $ACC_{d}$, $∆d_{dp}$ ($h_{1}-h_{2}$), $ACC_{T}$, and $RES_{T}$ (see Figure 9a,c,d,i,l, respectively) give rise to negligible values of $u_{∆V_{m}}$ that are always lower than 0.006 km/h.
- All the other effects, $∆d_{ct}$ (α), $∆d_{al}$ (β), $∆d_{co}$, $∆d_{te}$, $∆T_{rd}$, and $∆V_{SYNC}$ (see Figure 9e,f,g,h,m,n, respectively) give significant variation to $u_{∆V_{m}}$ that increase with the increasing of the speed value. Greater amplitude of $u_{∆V_{m}}$ are connected to $∆d_{al}$ and $∆V_{SYNC}$ with $u_{∆V_{m}}$ values that can reach more than 0.1 km/h even in this particularly favorable condition. This result is very important in order to understand both the limitations and the caution to be used in the applicability of this methodology in the calibration of high accuracy GNSS instrumentation.

### 5.3. Case Study 3

**Figure 10:**
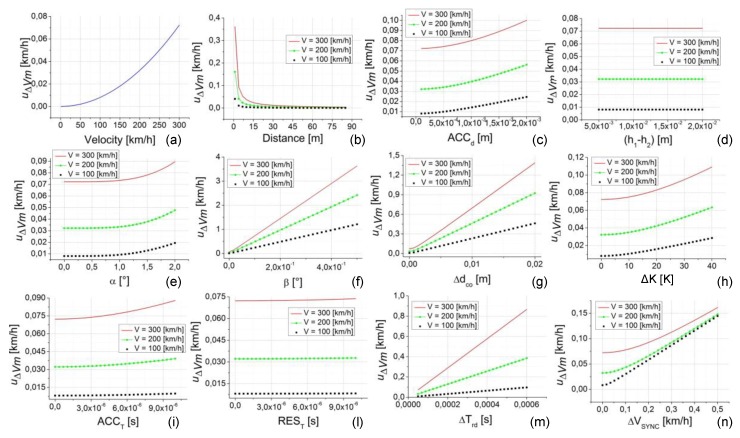
Uncertainty of the calibration method ($u_{∆V_{m}}$) versus the uncertainty contribution parameters. In each plot from (**a**–**n**) the independent variable is one parameter. The plots are relative to the case study 3 (d = 5 m, speed = 100, 200, and 300 km/h, all the other parameters set to their minimum value, as reported in Table 2).

The analysis of the third case study is reported in Figure 10. This case study was made in exactly the same conditions as case study 1 and 2 with the only difference being that the distance d was fixed at a value equal to 5 m (except that for the data shown in Figure 10b where d is the parameter under investigation so it varies in all its variability range). The value of d = 5 m was chosen as a representative value used in a typical application of the calibration setup under investigation [12,13].

Looking at Figure 10, it is easy to understand, as this is an intermediate situation between case studies 1 and 2. Therefore, similar considerations can be made with $u_{∆V_{m}}$ values that are never completely negligible (if not at the lower speed values). In particular, the parameters $∆d_{al}$ (β), $∆d_{co}$ and $∆T_{rd}$ (see Figure 10f,g,m, respectively) give significant variation to $u_{∆V_{m}}$ that increases with the increasing of the speed value.

Looking at Figure 10f, it is possible to note that the effect of $∆d_{al}$ that gives rise to a $u_{∆V_{m}}$ greater than 0.5 km/h already at the β values of about 0.1 °, reaching more than 3 km/h for the higher values of speed and β (this effect is connected to the methodology and instrumentation to be used for the calibration setup). Effects on $u_{∆V_{m}}$ up to about 1.3 km/h and 0.9 km/h can also be observed for $∆d_{co}$ (Figure 10g) and $∆T_{rd}$ (Figure 10m), respectively (these last effects are connected to the choice, the quality, and consequently, the cost of the photocells).

### 5.4. Case Study 4

**Figure 11:**
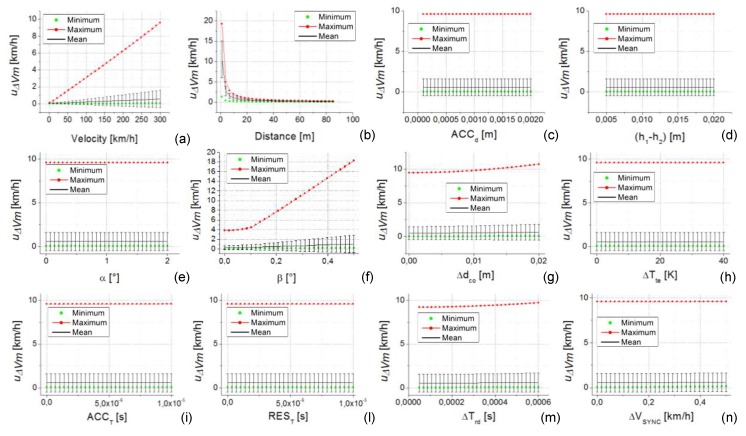
Uncertainty of the calibration method ($u_{∆V_{m}}$) versus the uncertainty contribution parameters. In each plot from (**a**–**n**) the independent variable is one parameter. The plots are relative to the case study 4 (all the parameters are randomly selected inside their own range of variability reported in Table 2—except the case (a) where the speed is fixed at 300 km/h). The black bars near the mean values represent the standard deviation of the $u_{∆V_{m}}$ calculated on the 1000 random repetitions.

The analysis of the fourth considered case study is reported in Figure 11. In this case, all the uncertainty contribution parameters are randomly selected inside their own range of variability reported in Table 2. Fixing the value of all parameters in this way, each single parameter has been varied inside its variability range to obtain the plots with respect to each one. The analysis is repeated several times, each time selecting a new set of random parameters. At the end of this procedure, the maximum, the minimum, the mean value, and the standard deviation of the obtained uncertainties $u_{∆V_{m}}$ were calculated and plotted in Figure 11 for a speed value of 300 km/h (considering 1000 random repetitions).

This case study allows us to highlight the contribution to the uncertainty of the calibration method ($u_{∆V_{m}}$) due to the cumulative effect of all uncertainty parameters randomly selected inside their variability range. In this way, a situation nearer to the real setup of the calibration method is analyzed. In fact, depending on the choice made in the selection of the methods for the setup realization and the instrumentation to be used (inside the characteristics shown in Table 2), the effect on the uncertainty of some parameters can be higher and the others can be lower.

In Table 3, a numeric summary of the results shown in Figure 11 is reported. In particular, the maximum, minimum, and mean of the obtained uncertainties $u_{∆V_{m}}$ versus the different uncertainty parameters is reported for all the three considered speed values (100 km/h, 200 km/h, and 300 km/h). Looking at Figure 11 and Table 3, a number of considerations can be made about the influence of the single parameters to the uncertainty $u_{∆V_{m}}$.

First, the values assumed by some influence parameters do not have effect on $u_{∆V_{m}}$ (the maximum, minimum, and mean values of $u_{∆V_{m}}$ that are substantially constant with respect to the variation of the considered parameter). The parameters that have effect on the $u_{∆V_{m}}$ are speed, d, β, and $∆d_{co}$, as shown in Figure 11a,b,f,g, respectively. While not visible in Figure 11n, there is also a variation of the minimum values of $u_{∆V_{m}}$ with $∆V_{SYNC}$ (see Table 3 last two rows). For all these parameters, the effect is an increase of the $u_{∆V_{m}}$ value increasing the parameter value (except d for which the effect, as expected, is the opposite). The variation of $u_{∆V_{m}}$ with these parameters is expressed on Table 3 by means of two rows of data corresponding to speed, d, β, $∆d_{co}$, and $∆V_{SYNC}$: In the first row, the minimum values in the second the maximum are reported. For instance, looking at Figure 11f, the maximum values (of the 1000 random repetitions) range from a minimum of 3.79 km/h to a maximum of 18.29 km/h, as reported in the first column of Table 3 in correspondence to the rows related to parameter β.

Secondly, $u_{∆V_{m}}$ is strictly connected to the values assumed by speed and d. This is visible by the great variations observed by all the considered $u_{∆V_{m}}$ quantities (max, min, and mean) with respect to these values.

Obviously, as shown in Table 4, the minimum values are obtained by a favorable set of random parameters connected primarily to high values of d and low values of both the speed and the other parameters. The smallest values of uncertainty are obtained with most of the parameters set to their minimum value (often zero). Conversely, the maximum values are obtained by an unfavorable set of random parameters (low values of d and high values of speed and the other parameters).

The maximum values (max) of $u_{∆V_{m}}$ are almost always greater than 9 km/h, 6 km/h and 3 km/h for the speed values 300 km/h, 200 km/h, and 100 km/h, respectively. At the same time, the mean values (mean) are almost always greater than 0.5 km/h, 0.3 km/h, and 0.2 km/h for the same speed values, respectively.

The values assumed by max and mean of $u_{∆V_{m}}$ give rise to an unsuitable methodology to calibrate high accuracy GNSS instrumentation that, as declared by the manufacturer, has measurement uncertainty lower than 0.06 km/h.

Taking into account the minimum values (min) of $u_{∆V_{m}}$, they are almost always lower than 0.05 km/h, 0.03 km/h, and 0.02 km/h (for the speed values 300 km/h, 200 km/h, and 100 km/h, respectively) giving rise to a possible suitability of this methodology to calibrate high accuracy GNSS instrumentation. As mentioned above, these min values of $u_{∆V_{m}}$ are connected to a favorable set of random parameters not always applicable even if a particular attention on the design of the calibration setup is taken. In fact, also considering this favorable set of parameters, there are cases where, if one of the parameter changes (d, β, $∆d_{co}$, $∆V_{SYNC}$), the min value of $u_{∆V_{m}}$ easily becomes greater than 0.06 km/h (the value of the uncertainty of the instrument to be calibrated).

Finally, the suitable values of $u_{∆V_{m}}$, reported in the first row of Table 3, cannot be applied for calibration purposes since they are obtained for speed values lower than 10 km/h, substantially making the calibration unnecessary.

The conditions of the case study 4 (1000 random repetitions) are repeated 20 times, obtaining similar results. Obviously, the obtained numeric values were slightly different, because the random choice of the sets of parameters, but the final consideration is always the same.

### 5.5. Case Study 5

The fifth considered case study was made in exactly the same manner as case study 4 with the only difference that the distance was fixed to a value of d = 5 m (it was chosen as representative value used in a typical application of the calibration setup under investigation [12,13]). Therefore, all the uncertainty contribution parameters are randomly selected inside their own range of variability reported in Table 2, while the distance is always fixed at d = 5 m; the tests were repeated for three different values of 100 km/h, 200 km/h, and 300 km/h.

Considering 1000 random repetitions, as for case study 4, the maximum, the minimum, and the mean value of the obtained uncertainties $u_{∆V_{m}}$ were calculated and are shown in Table 5.

Looking at the results, the first consideration is related to a remarkable reduction of the maximum (max) and a considerable increment of the minimum (min) values of $u_{∆V_{m}}$ due to the fact that, fixing the distance d, it cannot achieve the unfavorable values (near 1 m) and the favorable values (near 85 m), respectively.

In more detail, the maximum values (max) of $u_{∆V_{m}}$ are almost always greater than 3 km/h, 2 km/h, and 1 km/h for the speed values 300 km/h, 200 km/h, and 100 km/h, respectively. At the same time, the mean values (mean) are almost always greater than 2 km/h, 1 km/h, and 0.7 km/h for the same speed values, respectively.

The values assumed by the max and mean of $u_{∆V_{m}}$ give rise to an unsuitable methodology to calibrate GNSS instrumentation that has measurement uncertainty lower than 0.06 km/h.

Taking into account the minimum values (Min) of $u_{∆V_{m}}$, they are almost always (cases related to 200 km/h and 300 km/h) greater than 0.14 km/h, giving rise to an unsuitable methodology to calibrate GNSS instrumentation. Only in the cases where the speed is reduced to 100 km/h do the minimum (min) values of $u_{∆V_{m}}$ become comparable with 0.06 km/h (the uncertainty of the instrument to be calibrated).

Finally, the values of $u_{∆V_{m}}$, reported in the first row of Table 5, cannot be applied for calibration purposes since they are obtained for speed values lower than 10 km/h.

In conclusion, if the distance *d* is fixed to their typical values (5 m), the calibration of high accuracy GNSS instrumentation is, substantially, not feasible.

Even in case study 5, the conditions (1000 random repetitions) were repeated 20 times, obtaining the same results.

## 6. Conclusions

In this paper, the uncertainty contributions of a measurement setup, based on a couple of photocells, applied References [12,13] to calibrate the speed measured by the Global Navigation Satellite System (GNSS) and have been analyzed. The study has been performed in a speed range from 1 km/h to 300 km/h. After a detailed analysis of the uncertainty contribution due to the calibration method under investigation, the relationships between the uncertainty contributions and the calibration uncertainty due to the calibration method has been formulated. The uncertainty contributions have been studied and a variability range has been proposed to each one of them (with most of the minimum values selected equal to zero).

Comprehended in the five test cases, several analyses have been made, considering both fixed and random selections of the uncertainty contributions (inside their assigned variability range), analyzing more than 50,000 different combinations.

The first result is that some uncertainty contributions affect the total uncertainty more than others. For example, the uncertainty contribution related to the geometrical parameters connected to both the measurement methodology and setup (distance between the photocells, orthogonality of the two photocells lines respect to the road axis, the collimation of the laser beam) has a considerable effect on the total calibration uncertainty. Additionally, the speed value and the uncertainty contribution due to the synchronization between the measurements made by the standard instrument (on the road) and the instrument under calibration (on the testing vehicle) have a not negligible effect on the total calibration uncertainty.

Thus, depending on the desired level of calibration uncertainty, and on the desired level of the operative calibration complexity, it is possible to make the right choice about both the reference and the auxiliary instruments to be used for the calibration setup in order to meet the right trade-off between the technical constrains, satisfaction, and the costs.

The final important consideration is connected to the calibration of GNSS based instruments with accuracy declared by the manufacturers lower than 0.1 km/h (uncertainty lower than 0.06 km/h) where the good calibration practice request for a calibration uncertainty lower (1/2, 1/3, or 1/10) than the calibration of the instrument under calibration. The obtained results have shown as even making expensive and complex choices for the realization of the calibration setup (using the lower value of the uncertainty contributions) it is very hard to obtain uncertainty lower than 0.06 km/h especially at speed values greater than 150–200 km/h.

In conclusion, the analyzed measurement setup can be used for the calibration of high accuracy GNSS based instruments only considering distances between the photocells greater than 20 m and for speed values lower than 200 km/h. Even in these cases, particular attention has to be made in the choice of the other aspects (both instrumentations and methodologies) of the measurement setup.

## Figures and Tables

**Table 1:** Distance interval between two consecutive measurements made by the Global Navigation Satellite System (GNSS) receiver as function of the GNSS update rate and the vehicle speed.

		Distance Interval *d_i_* [m]					
Update Rate [Hz]		1	2	5	10	20	100
**Vehicle Speed *v_i_* [km/h]**	30	0.278	0.139	0.056	0.028	0.014	0.003
	100	27.778	13.889	5.556	2.778	1.389	0.278
	200	55.556	27.778	11.111	5.556	2.778	0.556
	300	83.333	41.667	16.667	8.333	4.167	0.833

**Table 2:** Variability ranges of the sources of uncertainty.

Parameter	Range		Units
	Min	Max	
Speed	1	300	km/h
d	1	85	m
$ACC_{d}$	0.1	2	mm
(h_1_ − h_2_)	0.5	2	cm
α	0	2	°
β	0	0.5	°
$∆d_{co}$	0	0.02	m
$∆d_{te}$	0	9	mm
$ACC_{T}$	10^−10^	10^−5^	s
$RES_{T}$	10^−10^	10^−5^	s
$∆T_{rd}$	5∙10^−5^	6∙10^−4^	s
$∆V_{SYNC}$	0	0.5	km/h

**Table 3:** Summary results for case study 4 considering 1000 random repetitions: Maximum (max), minimum (min), and mean (mean) of the obtained uncertainties $u_{∆V_{m}}$ versus the different uncertainty parameters.

		$u_{∆V_{m}}\;[\mathrm{km}/h]\;@300\;\mathrm{km}/h$			$u_{∆V_{m}}\;[\mathrm{km}/h]\;@200\;\mathrm{km}/h$			$u_{∆V_{m}}\;[\mathrm{km}/h]\;@100\;\mathrm{km}/h$		
		Max	Min	Mean	Max	Min	Mean	Max	Min	Mean
Speed	min	0.14	0.00048	0.074	0.14	0.00048	0.074	0.14	0.00048	0.074
	max	9.59	0.053	0.57	4.72	0.026	0.31	3.09	0.017	0.21
d	min	0.26	0.028	0.15	0.20	0.020	0.12	0.16	0.011	0.088
	max	19.25	1.341	10.52	12.83	0.764	6.87	6.42	0.315	3.39
$ACC_{d}$		9.60	0.053	0.57	6.26	0.034	0.39	3.09	0.017	0.21
h_1_ − h_2_		9.59	0.053	0.57	6.25	0.033	0.39	3.08	0.017	0.21
α		9.59	0.075	0.58	6.25	0.048	0.39	3.08	0.025	0.21
β	min	3.79	0.021	0.26	2.45	0.013	0.18	1.20	0.007	0.11
	max	18.29	0.221	1.02	12.15	0.148	0.69	6.06	0.074	0.36
$∆d_{co}$	min	9.45	0.020	0.52	6.15	0.011	0.35	3.03	0.008	0.20
	max	10.71	0.097	0.67	7.01	0.067	0.45	3.47	0.034	0.24
$∆d_{te}$		9.59	0.053	0.57	6.25	0.033	0.39	3.08	0.017	0.21
$ACC_{T}$		9.60	0.053	0.57	6.25	0.033	0.39	3.08	0.017	0.21
$RES_{T}$		9.59	0.053	0.57	6.25	0.033	0.39	3.08	0.017	0.21
$∆T_{rd}$		9.73	0.072	0.61	6.30	0.039	0.40	3.09	0.018	0.21
$∆V_{SYNC}$	min	9.59	0.024	0.56	6.25	0.013	0.36	3.08	0.004	0.18
	max	9.59	0.146	0.60	6.26	0.145	0.42	3.09	0.144	0.26

**Table 4:** Values of the set of random parameters associated to the maximum and minimum $u_{∆V_{m}}$ for case studies 4 and 5.

	Speed		d	$ACC_{d}$	h_1_ − h_2_	α	β	$∆d_{co}$	∆K	$ACC_{T}$	$RES_{T}$	$∆T_{rd}$	$∆V_{SYNC}$
	[Km/h]		[m]	[m]	[m]	[°]	[°]	[mm]	[K]	[μs]	[μs]	[μs]	[km/h]
**Case Study 4**	Min	*	70.6	0.0014	0.010	0.0048	0.0036	4.8	25.4	7.73	5.05	99.9	0.00031
	Max	*	1.3	0.00037	0.014	0.033	0.0060	6.6	24.6	6.18	7.98	512	0.074
**Case Study 5**	Min	*	5	0.0014	0.011	0.0033	0.00046	6.5	5.8	0.23	8.57	87.2	0.0015
	Max	*	5	0.0011	0.010	0.031	0.0087	19	9.3	4.41	2.66	397	0.12

* The speed values are fixed to the selected speed test (100 km/h, 200 km/h, 300 km/h).

**Table 5:** Summary results for the case study 5 considering 1000 random repetitions (for all parameters except d that is fixed to 5 m): Maximum (max), minimum (min), and mean (mean) of the obtained uncertainties $u_{∆V_{m}}$ versus the different uncertainty parameters.

		$u_{∆V_{m}}\;[\mathrm{km}/h]\;@300\;\mathrm{km}/h$			$u_{∆V_{m}}\;[\mathrm{km}/h]\;@200\;\mathrm{km}/h$			$u_{∆V_{m}}\;[\mathrm{km}/h]\;@100\;\mathrm{km}/h$		
		Max	Min	Mean	Max	Min	Mean	Max	Min	Mean
Speed	min	0.14	0.0023	0.074	0.14	0.0023	0.074	0.14	0.0023	0.074
	max	3.89	0.25	2.17	2.58	0.18	1.42	1.29	0.068	0.71
d		3.89	0.25	2.17	2.58	0.18	1.42	1.29	0.068	0.71
$ACC_{d}$		3.89	0.26	2.17	2.58	0.19	1.42	1.29	0.071	0.71
h_1_ − h_2_		3.89	0.25	2.17	2.58	0.18	1.42	1.29	0.068	0.71
α		3.89	0.25	2.17	2.58	0.18	1.42	1.29	0.069	0.71
β	min	1.61	0.12	0.90	1.00	0.06	0.55	0.49	0.031	0.26
	max	3.97	3.63	3.75	2.62	2.42	2.49	1.30	1.210	1.24
$∆d_{co}$	min	3.70	0.14	1.98	2.44	0.09	1.29	1.22	0.042	0.64
	max	3.95	1.39	2.49	2.61	0.93	1.64	1.30	0.464	0.82
$∆d_{te}$		3.89	0.25	2.17	2.58	0.19	1.42	1.29	0.068	0.71
$ACC_{T}$		3.89	0.25	2.17	2.58	0.18	1.42	1.29	0.068	0.71
$RES_{T}$		3.89	0.25	2.17	2.58	0.18	1.42	1.29	0.068	0.71
$∆T_{rd}$	min	3.84	0.14	2.09	2.56	0.10	1.39	1.29	0.058	0.70
	max	3.94	0.87	2.30	2.59	0.40	1.46	1.29	0.112	0.71
$∆V_{SYNC}$	min	3.88	0.21	2.17	2.57	0.12	1.42	1.28	0.058	0.70
	max	3.89	0.25	2.17	2.58	0.19	1.43	1.29	0.155	0.72

## References

[1] Szarmes M., Ryan S., Lachapelle G., Fenton P. (1997). DGPS high accuracy aircraft velocity determination using Doppler measurements. Proceedings of the International Symposium on Kinematic Systems in Geodesy, Geomatics and Navigation.

[2] Bevly D.M., Gerdes J.C., Wilson C. (2002). The use of GPS based velocity measurements for measurement of sideslip and wheel slip. Veh. Syst. Dyn..

[3] Al-Gaadi K.A. (2005). Testing the Accuracy of Autonomous GPS in Ground Speed Measurement. J. Appl. Sci..

[4] Townshend A.D., Worringham C.J., Stewart I.B. (2008). Assessment of speed and position during human locomotion using non differential GPS. Med. Sci. Sports Exerc..

[5] Gløersen Ø.N., Kocbach J., Gilgien M. (2018). Tracking Performance in Endurance Racing Sports: Evaluation of the Accuracy Offered by Three Commercial GNSS Receivers Aimed at the Sports Market. Front. Physiol..

[6] Beato M., Bartolini D., Ghia G., Zamparo P. (2016). Accuracy of a 10 Hz GPS unit in measuring shuttle velocity performed at different speeds and distances (5–20 M). J. Hum. Kinet..

[7] Keskin M., Sekerli Y.E., Kahraman S. (2017). Performance of two low-cost GPS receivers for ground speed measurement under varying speed conditions. Precis. Agric..

[8] Wang F., Zhang X., Huang J. (2008). Error analysis and accuracy assessment of GPS absolute velocity determination without SA. Geo Spat. Inf. Sci..

[9] Chalko T.J. (2007). High Accuracy speed measurement using GPS. NU J. Discov..

[10] Ding W., Wang J. (2011). Precise Velocity Estimation with a Stand-Alone GPS Receiver. J. Navig..

[11] Rezaali V., Ardalan A.A. (2016). Marine Current Meter Calibration Using GNSS Receivers, a Comparison with Commercial Method. Civ. Eng. J..

[12] Yeh T.K. (2015). Calibration of the GNSS Receivers—Methods, Results and Evaluation. Satellite Positioning.

[13] ISO (2017). General Requirements for the Competence of Testing and Calibration Laboratories.

[14] ILAC (2013). ILAC Policy on the Traceability of Measurement Results.

[15] European Parliament (2008). Regulation (EC) No 765/2008 of the European Parliament and of the Council of 9 July 2008 Setting out the Requirements for Accreditation and Market Surveillance Relating to the Marketing of Products and Repealing Regulation (EEC) No 339/93 (Text with EEA Relevance).

[16] ACCREDIA-Calibration Laboratories Department (2018). Requirements for the Accreditation of Calibration Laboratories.

[17] Petrovski I., Tsujii T., Perre J.M., Townsend B., Ebinuma T. (2010). GNSS Simulation: A User’s Guide to the Galaxy. Inside GNSS.

[18] Dong L., Ma C., Lachapelle G. Implementation and Verification of a Software-Based IF GPS Signal Simulator. Proceedings of the 2004 National Technical Meeting of The Institute of Navigation.

[19] Corbell P.M. (2000). Design and Validation of an Accurate GPS Signal and Receiver Truth Model for Comparing Advanced Receiver Processing Techniques. Master’s Thesis.

[20] Anthony K.B., Mirsky S.A. Validation of IEC 2400 GPS simulator for high dynamic RTK applications. Proceedings of the 57th Annual Meeting of the Institute of Navigation.

[21] Dyukov A. (2016). Development of an Electronic Speed Measurement System for Evaluating the Accuracy of GNSS Receivers and Statistical Analysis of Their Performance in Speed Measurements. Univers. J. Electr. Electron. Eng..

[22] Serrano L., Kim D., Langley R.B. A single GPS receiver as a real-time, accurate velocity and acceleration sensor. Proceedings of the ION GNSS 17th ITM.

[23] Bai Y., Sun Q., Du L., Yu M., Bai J. (2015). Two laboratory methods for the calibration of GPS speed meters. Meas. Sci. Technol..

[24] Bai Y., Sun Q., Du L., Yu M., Bai J. Calibration of GPS based high accuracy speed meter for vehicles. Proceedings of the International Symposium on Precision Engineering Measurement and Instrumentation.

[25] Ogle J., Guensler R., Bachman W., Koutsak M., Wolf J. (2002). Accuracy of Global Positioning System for Determing Driver Performance Parameters. J. Transp. Res. Board.

[26] Dyukov A., Choy S., Silcock D. (2015). Accuracy of Speed Measurements using GNSS in Challenging Environments. Asian J. Appl. Sci..

[27] Sathyamoorthy D., Shafii S., Amin Z.F.M., Jusoh A. (2015). Evaluation of the accuracy of global positioning system (GPS) speed measurement via GPS simulation. Def. S&T Tech. Bull..

[28] Du L., Sun Q., Cai C., Bai J., Fan Z., Zhang Y. (2018). A Vehicular Mobile Standard Instrument for Field Verification of Traffic Speed Meters Based on Dual-Antenna Doppler Radar Sensor. Sensors.

[29] Martucci A., Cerasuolo G., Petrella O., Laracca M. An Uncertainty Analysis for the Calibration of GNSS-Based Vehicle Speed Meters. Proceedings of the IEEE International Workshop on Metrology for Industry 4.0 and IoT (MetroInd4.0&IoT).

